# Tumor-associated neutrophils suppress pro-tumoral IL-17+ γδ T cells through induction of oxidative stress

**DOI:** 10.1371/journal.pbio.2004990

**Published:** 2018-05-11

**Authors:** Sofia Mensurado, Margarida Rei, Telma Lança, Marianna Ioannou, Natacha Gonçalves-Sousa, Hiroshi Kubo, Marie Malissen, Venizelos Papayannopoulos, Karine Serre, Bruno Silva-Santos

**Affiliations:** 1 Instituto de Medicina Molecular João Lobo Antunes (iMM), Faculdade de Medicina, Universidade de Lisboa, Lisboa, Portugal; 2 The Francis Crick Institute, London, United Kingdom; 3 Centre d'Immunologie de Marseille-Luminy, Aix-Marseille Université, Inserm, CNRS, Marseille, France; 4 Instituto Gulbenkian de Ciência, Oeiras, Portugal; National Cancer Institute, United States of America

## Abstract

Interleukin 17 (IL-17)–producing γδ T cells (γδ17 T cells) have been recently found to promote tumor growth and metastasis formation. How such γδ17 T-cell responses may be regulated in the tumor microenvironment remains, however, largely unknown. Here, we report that tumor-associated neutrophils can display an overt antitumor role by strongly suppressing γδ17 T cells. Tumor-associated neutrophils inhibited the proliferation of murine CD27^−^ Vγ6^+^ γδ17 T cells via induction of oxidative stress, thereby preventing them from constituting the major source of pro-tumoral IL-17 in the tumor microenvironment. Mechanistically, we found that low expression of the antioxidant glutathione in CD27^−^ γδ17 T cells renders them particularly susceptible to neutrophil-derived reactive oxygen species (ROS). Consistently, superoxide deficiency, or the administration of a glutathione precursor, rescued CD27^−^ Vγ6^+^ γδ17 T-cell proliferation in vivo. Moreover, human Vδ1^+^ γδ T cells, which contain most γδ17 T cells found in cancer patients, also displayed low glutathione levels and were potently inhibited by ROS. This work thus identifies an unanticipated, immunosuppressive yet antitumoral, neutrophil/ROS/γδ17 T-cell axis in the tumor microenvironment.

## Author summary

Tumors are infiltrated by many immune cells that influence many aspects of cancer progression and outcome, including tumor growth, invasion of healthy surrounding tissues, formation of metastasis, and response to treatments. Among tumor-infiltrating lymphocytes, γδ T cells play dual functions in the tumor milieu; whereas those that produce the antitumor cytokine interferon-γ are protective, their counterparts that make interleukin 17 (IL-17) support tumor growth. It is therefore critical to understand which mechanisms may limit IL-17–biased γδ T-cell responses. In this study, we unexpectedly found that IL-17^+^ γδ T cells express very low levels of the antioxidant, glutathione, and are very sensitive to reactive oxygen species (ROS), thus revealing their Achilles’ heel. Indeed, as ROS-producing neutrophils accumulate within tumors, they inhibit IL-17^+^ γδ T-cell proliferation and thereby suppress their pro-tumoral activities. We extended these findings, obtained in mouse models of cancer, to human γδ T cells and therefore believe that the modulation of local levels of oxidative stress may have important therapeutic implications.

## Introduction

A hallmark of solid tumors is their infiltration by immune cells that can either inhibit or promote tumor cell growth. Amongst such immune populations, γδ T cells are known to contribute to protective responses because of their potent ability to kill tumor cells and to produce cytokines like interferon gamma (IFN-γ) and tumor necrosis factor alpha (TNF-α) [1–5], which constitutes a solid basis for γδ T-cell–based cancer immunotherapy strategies [6]. In stark contrast, cumulative evidence indicates that interleukin 17 (IL-17)–producing γδ (γδ17) T cells promote tumor progression in several experimental models, including a genetic mouse model of pancreatic intraepithelial neoplasia [7]; transplantable models of subcutaneous fibrosarcoma, skin carcinoma, and colon cancer [8]; subcutaneous and intrahepatic hepatocellular carcinoma [9]; as well as intraperitoneal ovarian cancer [10]. In addition to contributing to primary tumor development and progression, recent reports revealed metastasis-promoting features of γδ17 T cells, both in a genetic mouse model of breast cancer metastasis [11] and in transplantable mouse models of lung metastasis [12]. Importantly, in human cancers, γδ17 T cells were also observed and associated with advanced stages of disease in colorectal and squamous cell skin tumors [13,14] and decreased survival of patients with gallbladder cancer [15]. Of note, the Vδ1^+^ subpopulation of human γδ T cells was reported to be a major source of IL-17 in colon cancer [13] and squamous cell skin cancer [14] patients and to promote inflammation-induced cancer progression [16].

The pro-tumoral function of γδ17 T cells was shown to result from either direct support of tumor cell survival, through the interleukin 6 (IL-6)–signal transducer and activator of transcription 3 (STAT3) axis [7,17], or indirect establishment of a prosperous environment for the tumor, especially through promotion of angiogenesis [8,15]. Moreover, part of these pro-tumoral effects occurs via recruitment/activation of myeloid cells. For instance, we have shown that γδ17 T cells accumulate in a mouse model of ovarian cancer and that they induce the mobilization of small peritoneal macrophages that express pro-inflammatory and pro-angiogenic mediators [10]. Other pro-tumoral myeloid subsets mobilized by γδ17 T cells include neutrophils [11] as well as myeloid-derived suppressor cells of both monocytic [9] and polymorphonuclear [13,18] lineages, which converge in the suppression of antitumor CD8^+^ T-cell responses. Thus, γδ17 T cells have been extensively shown to interact with myeloid cells that counteract tumor immune surveillance, and instead promote cancer progression.

This notwithstanding, a large-scale analysis of thousands of tumor samples from 39 cancer types indicated that γδ T cells are globally associated with a good prognosis [19], which may suggest that γδ17 T-cell responses are often limited by as yet unknown mechanisms. In fact, very little is known about the regulatory pathways that may control γδ17 T cells in cancers. We thus undertook to determine the cellular and molecular mechanisms controlling γδ17 T-cell responses in the tumor microenvironment.

In mice, γδ17 T cells are comprised in discrete thymic and peripheral CD27^−^ γδ T-cell compartments [20] and can be further subdivided into two main subpopulations expressing either Vγ4^+^ or Vγ6^+^ T-cell receptors (TCRs) [21]. These subsets have distinct developmental requirements in the thymus [22,23] and different homeostasis and dynamics in peripheral tissues [21]. Namely, whereas Vγ4^+^ γδ17 T cells typically populate secondary lymphoid organs (from which they can be mobilized upon challenge), their Vγ6^+^ counterparts leave the fetal thymus to become tissue-resident, long-lived, and self-renewing cells that respond in situ [24–26]. This is particularly relevant in tissues where Vγ6^+^ γδ17 T cells are abundant, such as the dermis, tongue, lung, liver, uterus, and peritoneal cavity [21]. Interestingly, CD27^−^ Vγ6^+^ γδ17 T cells proliferated extensively in the peritoneal cavity following the transplantation of ID8 ovarian cancer cells, thereby constituting the major source of pro-inflammatory and pro-angiogenic IL-17 that promoted tumor cell growth [10].

Following that study, we have investigated the contribution of γδ17 T cells to different tumor types developing in the same environment. Unexpectedly, we found that pro-tumoral γδ17 T cells failed to respond to discrete tumor challenges due to neutrophil-mediated suppression, which therefore limited tumor growth. We went on to dissect the molecular mechanisms underlying this unanticipated neutrophil/γδ17 T-cell cross talk in experimental mouse models of cancer and found an exquisite sensitivity of γδ17 T cells to reactive oxygen species (ROS)-induced oxidative stress in the tumor microenvironment.

## Results

### Tumor-associated neutrophils suppress IL-17–producing γδ T-cell responses

**Fig 1:**
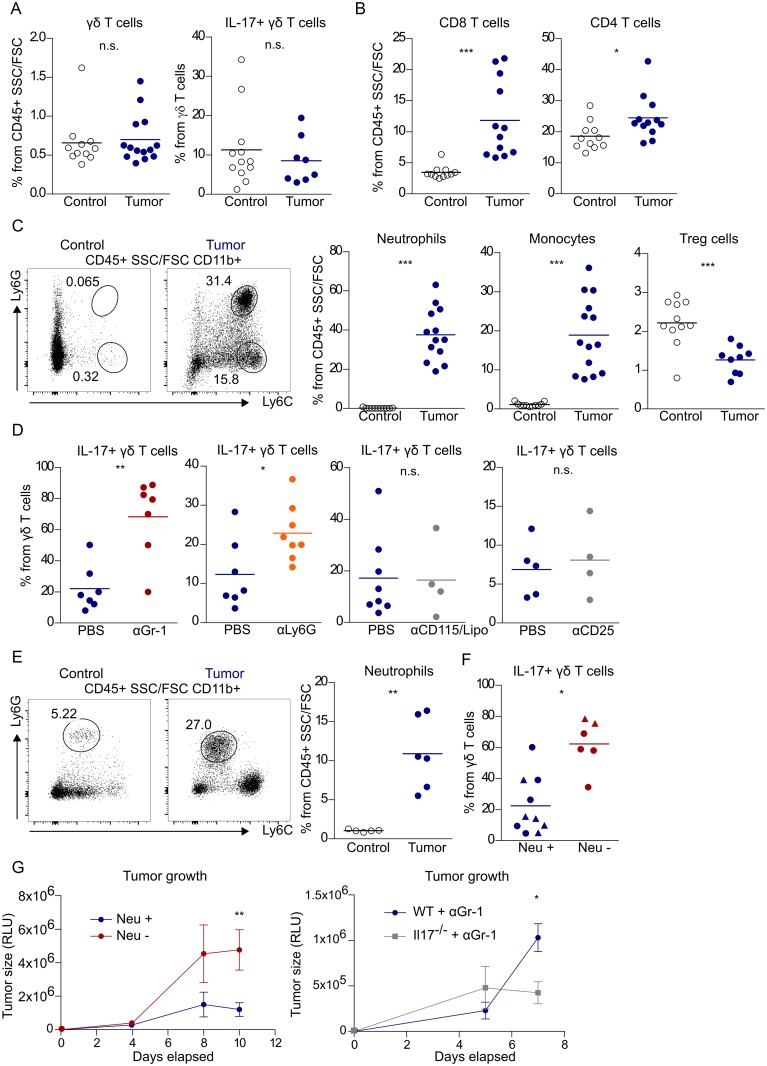
Tumor-associated neutrophils suppress γδ 17 T-cell responses. Frequency of **(A)** total and IL-17^+^ γδT cells and **(B)** CD8^+^ and CD4^+^ T cells in the PEC of tumor-free and B16-F0 tumor-bearing mice. Data were pooled from four different experiments. **(C)** Representative FACS plots and summary of neutrophil, monocyte, and Treg cell frequency in the PEC of tumor-free and B16 tumor-bearing mice. Data were pooled from four independent experiments. **(D)** Frequency of IL-17^+^ γδ T cells in B16 tumor–bearing mice injected with vehicle (PBS) or mAb αGr-1, αLy6G, αCD115 + clodronate liposomes, and αCD25. **(E)** Representative FACS plots and frequency of neutrophils in tumor-free liver and within Hepa 1–6 intrahepatic tumor developed in C57BL/6 mice and **(F)** frequency of IL-17^+^ γδ T cells within Hepa 1–6 tumors developed in mice deficient/depleted for neutrophils (Neu −) or respective controls (Neu +). Red and blue circles represent αGr-1 mAb-treated or PBS-treated C57BL/6 mice, respectively, whereas red and blue triangles represent *Genista* homozygous or littermate controls, respectively. **(G)** Left: intrahepatic Hepa 1–6 tumor growth in mice with (heterozygous littermate control, *n* = 10) and without (*Genista* homozygous, *n* = 4) mature neutrophils. Data were pooled from two independent experiments. Right: intrahepatic Hepa 1–6 tumor growth in C57Bl/6J WT (*n* = 5) and *Il17*^−/−^ (*n* = 5) mice treated with αGr-1. Data presented as mean ± SEM. Statistical analysis was performed using Student *t* test or Mann-Whitney test. Data are provided in S1 Data. Lipo, clodronate liposome; mAb, monoclonal antibody; PEC, peritoneal exudate cell; RLU, relative luminescence units; Treg, regulatory T; γδ17, IL-17–producing γδ T cell.

This study initiated with an unexpected finding upon implantation of the B16-F0 cell line in the peritoneal cavity. In stark contrast to our previous observations with ID8 tumors [10], B16-F0 (simplified to B16) challenge did not increase the frequency of total γδ T cells or γδ17 T cells in the peritoneal cavity when compared to tumor-free controls (Fig 1A), while CD8^+^ and CD4^+^ T cells accumulated significantly (Fig 1B). We thus considered the possibility of γδ17 T cells being selectively inhibited by another immune cell population and examined leukocyte subsets previously associated with T-cell suppression. Interestingly, we found striking amounts of neutrophils in the peritoneal cavity of B16-bearing but not ID8-bearing mice (S1A Fig), thus segregating with the lack (Fig 1A) or presence [10] of γδ17 T-cell responses, respectively. In fact, upon B16 tumor challenge, both neutrophils (CD11b^+^Ly6G^+^Ly6C^int^) and monocytes (CD11b^+^Ly6G^-^Ly6C^+^) accumulated, respectively, 40- and 20-fold within the leukocyte infiltrate (CD45^+^ cells) (Fig 1C). Although regulatory T (Treg) cells decreased in frequency (Fig 1C), we nonetheless assessed their impact, in parallel with that of myeloid cells, on γδ17 T cells, through depletion strategies using anti-CD25 monoclonal antibody (mAb) that targets Treg cells, anti-Gr1 and anti-Ly6G mAbs that target neutrophils, or anti-CD115/clodronate-liposomes that target monocytes and macrophages. Of note, these approaches were very efficient at depleting the corresponding target leukocyte subsets (S2A Fig). Critically, only neutrophil depletion resulted in an increased frequency of IL-17^+^ γδ T cells in tumor-bearing mice (Fig 1D). Given that ID8 promoted the accumulation of IL-17^+^ γδ T cells in the peritoneal cavity [10], whereas in B16-bearing mice, the mobilization of neutrophils inhibited γδ17 T-cell responses, we questioned what would happen in animals bearing both tumor types. We found that neutrophil depletion still led to a marked increase in IL-17^+^ γδ T cells in ID8+B16-bearing mice (S1B Fig), thus suggesting that neutrophil-mediated inhibition is a dominant phenomenon.

We then aimed to validate and extend our findings to an orthotopic tumor model, and selected a hepatocellular carcinoma model (Hepa 1–6) in which tumor growth is increased in the presence of IL-17 [9]. We implanted the Hepa 1–6 cell line directly in the liver of C57BL/6 mice and analyzed the immune infiltrate. Similarly to the B16 model, the frequency of neutrophils increased significantly in the hepatic tumor within the hematopoietic infiltrate (CD45^+^ cells) compared to the tumor-free liver tissue (Fig 1E). We next depleted neutrophils using the anti-Gr-1 mAb and also used a genetically neutropenic mouse strain, *Genista*, which, because of a point mutation in the transcription factor growth factor independence 1 (*Gfi1*), lacks mature neutrophils (in the periphery and in the bone marrow) without impacting on lymphopoiesis nor on T- and B-Cell functions [27,28]. Consistently, tumor-bearing homozygous *Genista* mice displayed low frequencies of neutrophils, and the few remaining tumor-associated neutrophils expressed lower levels of the maturation markers, Ly6G and CD11b, when compared to littermate heterozygous controls (S2B Fig). Importantly, neutrophil depletion or deficiency also led to a robust increase in IL-17^+^ γδ T cells in the intrahepatic Hepa 1–6 model (Fig 1F). Moreover, we observed a 5-fold increase in tumor load in the homozygous *Genista* mice compared to their littermate controls (Fig 1G, left panel). This was in line with the reduced tumor growth of neutrophil-depleted *Il17*^−/−^ mice compared to neutrophil-depleted wild-type (WT) mice (Fig 1G, right panel) and supported our hypothesis that neutrophils limit tumor growth at least in part by inhibiting IL-17 production in the tumor microenvironment.

Along the same lines, the proportion of IL-17–producing cells (within CD45^+^ leukocytes) was increased upon neutrophil depletion/deficiency in both tumor models, while the frequency of IFN-γ–producing cells remained unchanged (S3A Fig). Importantly, the contribution of γδ T cells to IL-17 producers upon neutrophil depletion clearly outcompeted that of CD4^+^ T cells, for there were around 3-fold more IL-17^+^ γδ than IL-17^+^ CD4^+^ T cells (S3B Fig, left panels), and the IL-17 mean fluorescence intensity (MFI) was consistently higher in γδ compared to CD4^+^ T cells (S3B Fig, right panels). Taken together, these data suggest that neutrophils suppress tumor growth by inhibiting the major IL-17–producing population in the tumor niche, γδ17 T cells.

### Neutrophils selectively inhibit the proliferation of IL-17–producing CD27^−^ Vγ6^+^ T cells

**Fig 2:**
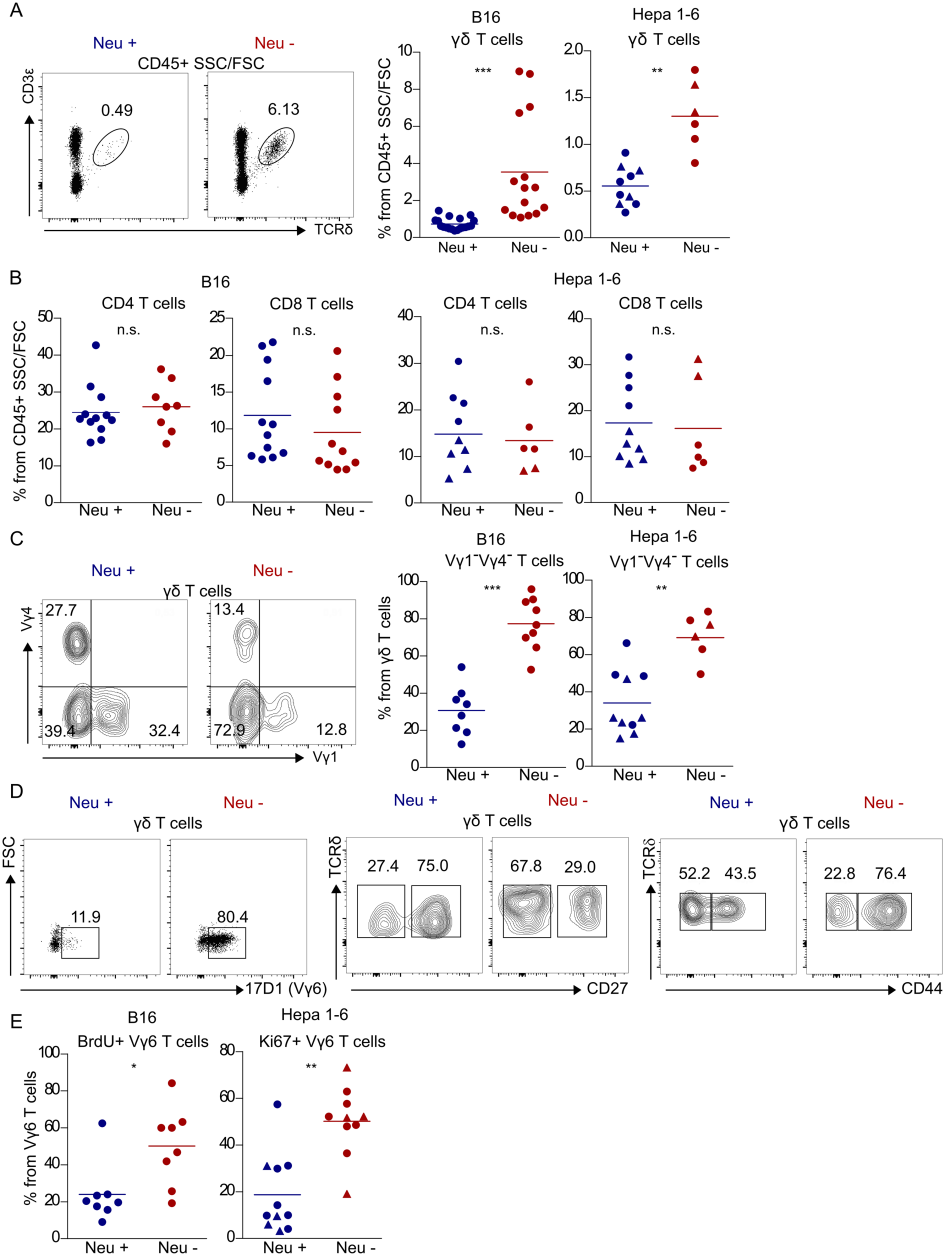
Neutrophils selectively inhibit the proliferation of Vγ6+ γδ T cells. Representative FACS plots and/or frequency (gated on CD45^+^ lymphocytes) of **(A)** γδ T cells, **(B)** CD4^+^ and CD8^+^ T cells, and **(C)** Vγ1^−^Vγ4^−^ γδ T cells (gated on γδ T cells) in intraperitoneal B16 or intrahepatic Hepa 1–6 tumors, developed in mice deficient/depleted for neutrophils (Neu −) or respective controls (Neu +). Red and blue circles represent αGr-1 mAb-treated or PBS-treated C57BL/6 mice, respectively, whereas red and blue triangles represent *Genista* homozygous or littermate controls, respectively. Data were pooled from two (Hepa 2–6) and three to five (B16) independent experiments. **(D)** Representative FACS plots of γδ T-cell phenotype in PBS− (Neu +) or αGr-1 (Neu −) mAb-treated B16 tumor–bearing mice. **(E)** Frequency of BrdU^+^ Vγ6^+^ T cells in B16 tumor–bearing mice and of Ki67^+^ Vγ6^+^ T cells in Hepa 1–6 tumor–bearing mice at days 9 and 21 post–tumor inoculation, respectively. Statistical analysis was performed using Student *t* test or Mann-Whitney test. Data are provided in S1 Data. BrdU, bromodeoxyuridine; mAb, monoclonal antibody; TCR, T-cell receptor.

Given that the ablation of neutrophils led to an increase in IL-17–producing γδ T cells, we investigated which γδ17 T-cell subset was affected and how—i.e., the cellular mechanism of suppression. In both tumor models, the absence of neutrophils provoked an increase in the total proportion of γδ T cells (Fig 2A) but had no effect on CD8^+^ or CD4^+^ T cells (Fig 2B). Neutrophils particularly affected γδ T cells negative for both Vγ1 and Vγ4 TCR chains, because these became dominant upon neutrophil depletion/deficiency (Fig 2C). By using the staining protocol that combines GL3 and 17D1 mAbs [10,29], we confirmed that the majority of these cells expressed the Vγ6 TCR chain (Fig 2D, left panel) while also mostly displaying a CD27^−^ CD44^+^ phenotype (Fig 2D, middle and right panels) that tightly associates with γδ17 T cells [20,30–32]. Importantly, we found that neutrophils dampened Vγ6^+^ T cells in vivo through inhibition of proliferation (Fig 2E) and not by inducing apoptosis or impairing their recruitment from secondary lymphoid organs (S4 Fig). In particular, we observed substantially increased 5-bromodeoxycytidine (BrdU) incorporation and higher proportions of Ki67^+^ Vγ6^+^ T cells in both *Genista* and in neutrophil-depleted mice (Fig 2E). These results indicate that neutrophils can selectively and potently inhibit CD27^−^ Vγ6^+^ T-cell proliferation in in vivo tumor models.

### Tumor-associated neutrophils inhibit CD27^−^ Vγ6^+^ T-cell proliferation via ROS production

**Fig 3:**
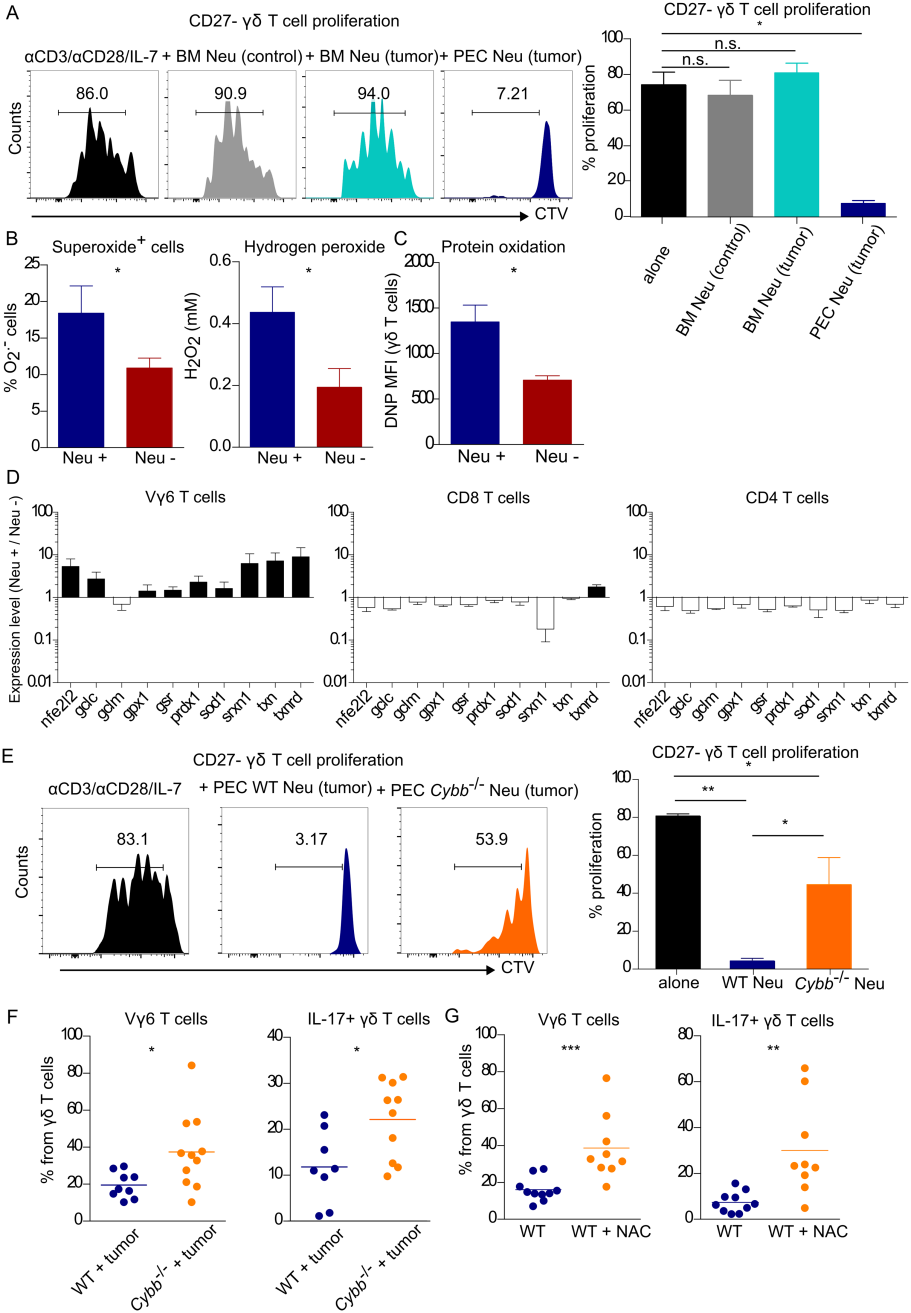
Tumor-associated neutrophils inhibit CD27^−^ Vγ6+ γδ T-cell proliferation by inducing oxidative stress. **(A)** Representative histograms and summary of in vitro CD27^−^ γδ T-cell proliferation cultured alone (*n* = 13), in the presence of neutrophils from BM of B16 tumor–free (*n* = 3) or tumor–bearing mice (*n* = 5), or with neutrophils from the PEC of B16 tumor–bearing mice (*n* = 7). Data were pooled from four independent experiments. **(B)** Total superoxide-positive cells in B16 tumor–bearing mice depleted (αGr-1 mAb, Neu −, *n* = 13) or not (Neu +, *n* = 8) for neutrophils. Data were pooled from three independent experiments. Total hydrogen peroxide levels in peritoneal supernatants of B16 tumor–bearing mice depleted (αGr-1 mAb, Neu −, *n* = 8) or not (Neu +, *n* = 7) for neutrophils. Data are representative of two independent experiments. **(C)** Protein oxidation assessed by flow cytometry in total γδ T cells from neutrophil-sufficient and neutrophil-depleted B16 tumor–bearing PEC. **(D)** Gene expression of oxidative stress–related genes in Vγ6^+^ T cells, CD4^+^, and CD8^+^ T cells sorted from B16 tumor–bearing PEC (Neu +), relative to the same populations sorted from neutrophil-depleted B16 tumor–bearing PEC (Neu −), normalized to *Hprt*. **(E)** Representative histograms and summary of in vitro CD27^−^ γδ T-cell proliferation, cultured alone or in the presence of neutrophils from the PEC of C57Bl/6J or *Cybb*^−/−^ (*Nox2*^−/−^) B16 tumor–bearing mice (*n* = 4, each). **(F)** Frequency of Vγ6^+^ and IL-17^+^ γδ T cells in PEC of C57Bl/6J and *Cybb*^−/−^ (*Nox2*^−/−^) B16 tumor–bearing mice, 13 days post–tumor inoculation. Data were pooled from two independent experiments. **(G)** Frequency of Vγ6^+^ T cells and IL17^+^ γδ T cells in PEC of C57Bl/6J B16 tumor–bearing mice, treated with PBS or NAC. Data were pooled from two independent experiments. Statistical analysis was performed using two-way ANOVA followed by Tukey HSD post hoc test, Student *t* test, or Mann-Whitney test. Data are provided in S1 Data. BM, bone marrow; CTV, cell trace violet; *Cybb*, cytochrome B(−245), β subunit; DNP, dinitrophenyl; Gclm, glutamate-cysteine ligase modifier subunit; Gcl, glutamate-cysteine ligase; Gpx, glutathione peroxidase; Gsr, glutathione reductase; *Hprt*, hypoxanthine-guanine phosphoribosyltransferase; mAb, monoclonal antibody; MFI, mean fluorescence intensity; NAC, N-acetylcysteine; *Nfe2l2*, nuclear factor, erythroid 2 like 2; *Nox2*, NADPH oxidase 2; PEC, peritoneal exudate cells; *Prdx*, peroxiredoxin; *Sod1*, superoxide dismutase 1; *Srxn1*, sulfiredoxin-1; *Txn*, Thioredoxin; *Txnrd*, Thioredoxin reductase.

Next, we dissected the molecular mechanism by which neutrophils suppressed γδ17 T cells using the B16-F0 intraperitoneal mouse model, because it allowed efficient purification of significant numbers of neutrophils from the PEC of tumor-bearing mice. In addition, we employed in vitro co-cultures to assess the direct impact of neutrophils on γδ17 T cells, in the absence of other cell types. We co-cultured purified neutrophils and CD27^−^ γδ T cells that were induced to proliferate in vitro via stimulation with anti-CD3 and anti-CD28 mAbs [33]. We found that the proliferation of CD27^−^ γδ T cells was inhibited when cultured with tumor-associated neutrophils, but not with bone marrow–derived neutrophils from either tumor-bearing or tumor-free mice (Fig 3A). These results show that the tumor microenvironment endows neutrophils with their suppressive phenotype and that tumor-associated neutrophils are sufficient to exert direct inhibition on CD27^−^ γδ T-cell proliferation. Moreover, consistent with the fact that IFN-γ+ cells (S3A Fig), CD4+, and CD8+ T cells (Fig 2B) are not affected by neutrophil depletion in vivo, we found that neutrophils from tumor-bearing mice preferentially impacted the in vitro proliferation of CD27^−^ γδ T cells when compared to CD27^+^ γδ, CD4^+^, and CD8^+^ T cells (S5A Fig). One mechanism employed by neutrophils for immunosuppression is the production of ROS [34]. We thus analyzed ROS in peritoneal cells of tumor-bearing mice depleted or not for neutrophils. Neutrophil depletion reduced the percentages of superoxide-positive cells (as assessed by dihydroethidium staining) as well as the levels of hydrogen peroxide (Fig 3B), indicating that neutrophils were a major source of ROS in vivo. Moreover, γδ T cells from the peritoneal cavity of B16 tumor–bearing mice exhibited increased protein oxidation levels when compared to the same population in neutrophil-depleted B16 tumor–bearing mice (Fig 3C), suggesting that these cells are under oxidative stress in the presence of neutrophils. Consistent with this, the expression of enzymes or regulator genes involved in ROS scavenging was higher in Vγ6^+^ T cells from neutrophil-sufficient compared to neutrophil-depleted tumor-bearing mice. This indicates that Vγ6^+^ T cells are actively responding to oxidative damage, unlike CD4^+^ and CD8^+^ T cells, which are largely unchanged by the presence of neutrophils in the tumor microenvironment (Fig 3D).

To directly test the role of ROS-induced oxidative stress in the inhibition of γδ17 T cells, we used a cytochrome B(−245), β subunit (*Cybb)*^−/−^ mouse strain that lacks the enzyme NADPH oxidase 2 (NOX2), which catalyzes the conversion of molecular oxygen to superoxide. We purified neutrophils from the peritoneal cavity of tumor-bearing *Cybb*^−/−^ or WT mice and co-cultured them with anti-CD3/CD28-stimulated CD27^−^ γδ T cells. Whereas WT neutrophils drastically inhibited the proliferation of CD27^−^ γδ T cells, the latter were able to divide in the presence of *Cybb*^−/−^ neutrophils, albeit not as efficiently as in the complete absence of neutrophils (Fig 3E). Notably, CD27^−^ γδ T-cell proliferation was also restored in co-cultures with WT neutrophils when these were supplemented with catalase in a dose-dependent manner (S5B Fig). Critically, we validated these findings in vivo upon establishment of B16 tumors in *Cybb*^−/−^ (or WT) mice, as we found that Vγ6^+^ and IL-17^+^ γδ T cells accumulated to significantly higher levels in *Cybb*^−/−^ than in control mice (Fig 3F).

As a corollary to our working model, we tested the impact of the in vivo administration of a well-established antioxidant, N-acetylcysteine (NAC), as a potential gain-of-function approach. Indeed, NAC treatment was sufficient to lead to an accumulation of Vγ6^+^ and IL-17–producing γδ T cells in the peritoneal cavity of tumor-bearing mice (Fig 3G). Taken together, these results demonstrate that tumor-associated neutrophils potently suppress the proliferation of CD27^−^ Vγ6^+^ γδ17 T cells via ROS-mediated induction of oxidative stress.

### Low glutathione expression renders CD27^−^ γδ T cells highly susceptible to ROS-mediated suppression

**Fig 4:**
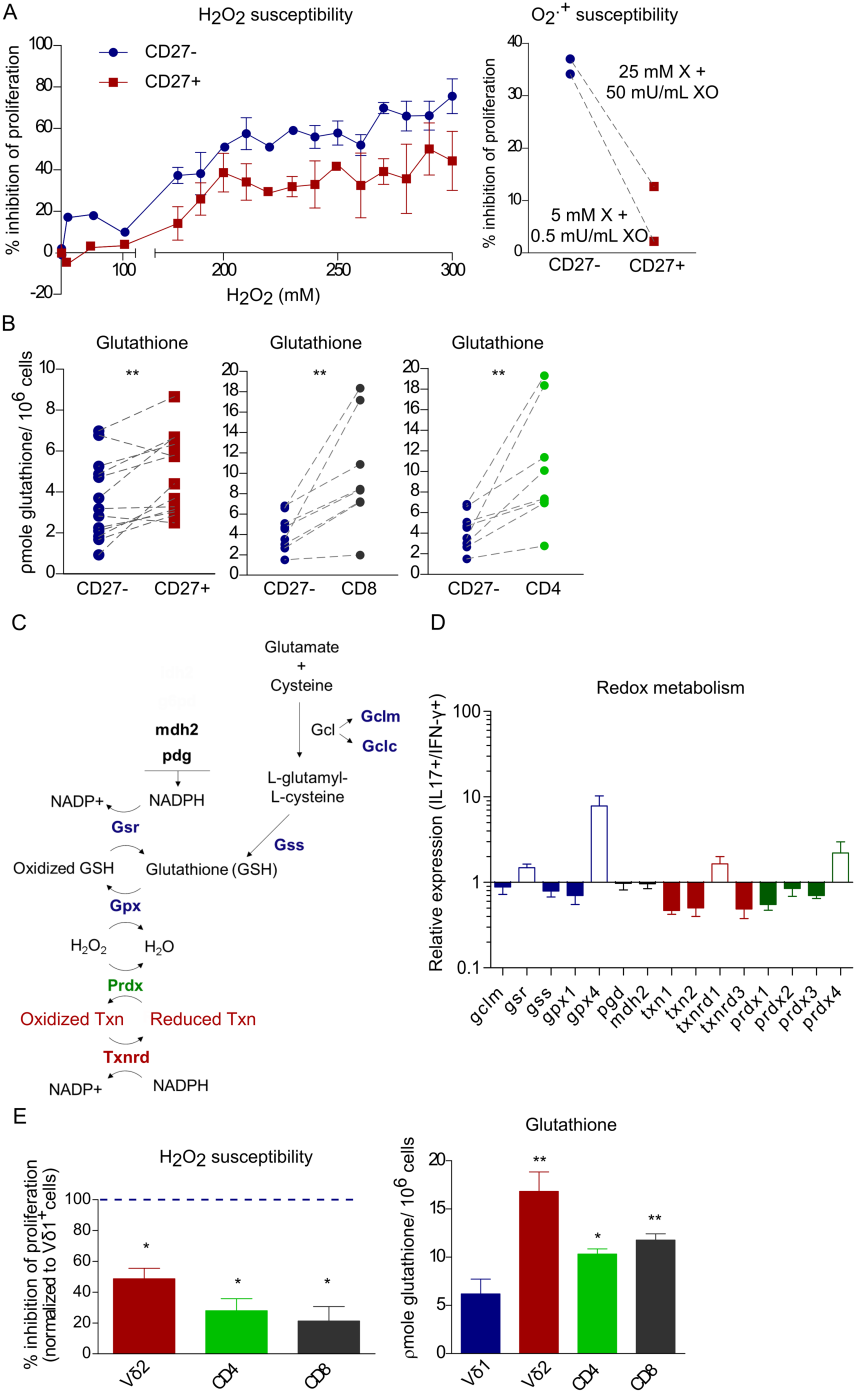
Murine CD27^−^ γδ T cells and human Vδ1^+^ γδ T cells express low levels of glutathione and are highly susceptible to ROS. **(A)** FACS-sorted CD27^−^ and CD27^+^ γδ T cells were stimulated and proliferation was assessed by CTV dilution, with increasing concentrations of H_2_O_2_ (left, *n* = 2–3) or with different concentrations of the superoxide-generating system X/XO, right. **(B)** Total glutathione levels in CD27^−^ γδ, CD27^+^ γδ, CD8^+^, and CD4^+^ T cells sorted from spleen and lymph nodes of tumor-free mice. Dotted lines link subsets from the same mouse. **(C)** Schematic representation of enzymes involved in redox metabolism. **(D)** Expression of redox-related genes in IL-17^+^ γδ T cells relative to IFN-γ^+^ γδ T cells at steady state, normalized to *Hprt* or *β2microglobulin*. **(E)** FACS-sorted Vδ1^+^, Vδ2^+^, CD8^+^, and CD4^+^ T cells (from buffy coats of healthy donors) were stimulated for 6 days in the presence of H_2_O_2_ (*n* = 4) and proliferation was assessed by CTV dilution, left. Total glutathione levels in Vδ1^+^, Vδ2^+^, CD4^+^, and CD8^+^ T cells (*n* = 5) sorted from buffy coats of healthy donors, right. Statistical analysis was performed Wilcoxon-matched-pairs signed rank test, Mann-Whitney test, and two-way ANOVA, followed by Tukey HSD post hoc test. Data are provided in S1 Data. CTV, cell trace violet; Gcl, glutamate-cysteine ligase; Gclc, glutamate-cysteine ligase catalytic subunit; Gclm, glutamate-cysteine ligase modifier subunit; Gpx, glutathione peroxidase; GSH, glutathione; Gsr, glutathione reductase; Gss, glutathione synthetase; *Hprt*, hypoxanthine-guanine phosphoribosyltransferase; Mdh2, malate dehydrogenase 2; Pgd, Phosphogluconate dehydrogenase; Prdx, Peroxiredoxin; ROS, reactive oxygen species; Txn, Thioredoxin; Txnrd, Thioredoxin reductase; X/XO, xanthine/xanthine oxidase.

To understand why Vγ6^+^ CD27^−^ γδ T cells were especially affected by neutrophil-derived ROS, we assessed the effect of increasing concentrations of hydrogen peroxide (H_2_O_2_) and superoxide (O_2_^.-^) (generated by the xanthine/xanthine oxidase system) on the proliferation of CD27^−^ and CD27^+^ γδ T-cell subsets in vitro. Both hydrogen peroxide and superoxide inhibited γδ T-cell proliferation, but CD27^−^ cells were clearly more susceptible than their CD27^+^ counterparts (Fig 4A). These results led us to hypothesize that CD27^−^ γδ17 T cells might have lower capacity to detoxify ROS than CD27^+^ γδ T cells (or other T-cell subsets). Moreover, as γδ17 T cells expanded upon in vivo administration of NAC (Fig 3G), and this acts as a precursor to glutathione, we analyzed this major intracellular antioxidant and found significantly reduced basal levels in CD27^−^ γδ17 T cells when compared to CD27^+^ γδ T cells, as well as CD8^+^ and CD4^+^ T cells (Fig 4B). This may explain why neutrophil-derived ROS selectively impacted on CD27^−^ γδ17 T-cell proliferation (Fig 1A and 1D) compared to CD27^+^ γδ T cells (S3A Fig), CD8^+^ or CD4^+^ T cells (Fig 1B) in neutrophil-rich tumor models. Consistent with this, we found that several enzymes or antioxidants involved in ROS detoxification (Fig 4C) were selectively down-regulated in IL-17^+^ γδ T cells compared to IFN-γ^+^ γδ T cells (Fig 4D). For example, *Gclm*, the gene that encodes for one of the subunits glutamate-cysteine ligase (the first rate limiting step of glutathione production), as well as *Gss*, the gene that encodes for glutathione synthetase, were expressed less in IL-17^+^ T cells, which may explain the low glutathione pool in CD27^−^ γδ T cells. Most other antioxidants, such as thioredoxins and peroxiredoxines, were also lower in IL-17^+^ γδ T cells. Altogether, this supports that differences in redox metabolism make γδ17 T cells more sensitive to oxidative stress than γδIFN-γ T cells.

Finally, we questioned whether this pattern of differential expression of glutathione and susceptibility to ROS also applied to human T-cell subsets. We found that Vδ1^+^ γδ T cells, the main γδ T-cell subset associated with IL-17 production in human tumors [13,14,16], were also profoundly affected by the presence of H_2_O_2_, in contrast with their Vδ2^+^ γδ, CD8^+^, and CD4^+^ T-cell counterparts (Fig 4E, left). Consistently, Vδ1^+^ T cells also expressed significantly lower levels of glutathione (Fig 4E, right). Altogether, these data strongly suggest that murine CD27^−^ γδ17 T and human Vδ1^+^ T cells are particularly susceptible to ROS-mediated suppression because of their low basal glutathione levels, thus providing novel cues on how to limit their cancer-promoting functions in the tumor microenvironment.

## Discussion

γδ17 T cells are known to enhance neutrophil mobilization in the context of several infections and also in response to tumors [6,21,35]. Moreover, a positive feedback loop between neutrophil-derived IL-1β and IL-17 responses [36] and γδ17 T cells [37] has been suggested. By contrast, here we show that neutrophils inhibit γδ17 T cells, thus revealing a dynamic and multifaceted cross talk between these cell types in the tumor microenvironment. While the circumstances that dictate positive versus negative interactions remain unclear, the latter have been documented in other immune contexts. For instance, neutrophil depletion in a protective model of pulmonary cryptococcosis [38] or in an experimental mouse model of human metapneumovirus infection [39] resulted in increased IL-17 production by γδ T cells, but underlying molecular mechanisms were not identified. Provocatively, neutrophils may even act as important “rheostat” of γδ17 T-cell homeostasis, because mice deficient for either C-X-C chemokine receptor type 2 (*Cxcr2*) or integrin beta chain-2 (CD18; *Itgb2*), which are characterized by low neutrophil counts in tissues, show increased tissue-resident γδ17 T cells [40,41].

A dual role for neutrophils in cancer has been suggested [42–46], and as a result, neutrophil depletion can either reduce [42,47–51] or increase [52–55] tumor burden. Within the tumor niche, neutrophils are often associated with cancer progression, namely through promotion of angiogenesis or suppression of antitumor effector lymphocytes. Thus, tumor-associated neutrophils can produce large amounts of matrix metallopeptidase 9 (MMP-9), which remodels the extracellular matrix; promotes the release of pro-angiogenic vascular endothelial growth factor (VEGF) [47,56]; and inhibits CD8^+^ T-cell functions via secretion of IL-10 [57], arginase 1 (which degrades extracellular arginine) [58], or reactive nitrogen species [11]. In fact, Coffelt and colleagues recently proposed, in a transplantable model K14cre;Cdh1F/F;Trp53F/F (KEP) of mammary tumor-bearing mice, a link between Vγ4^+^ γδ17 T cells and neutrophils that led to inducible nitric oxide synthase (iNOS)-dependent suppression of cytotoxic CD8^+^ T cells and promoted lung metastases [11].

By contrast, in the peritoneal B16 and intrahepatic Hepa 1–6 tumor models, neutrophils inhibited γδ17 T cells, but not CD8^+^ T cells, through NOX2-dependent ROS production. These discordant actions of neutrophils in different tumor models may rely on their relative ROS levels and differential impact on T-cell subsets. As suggested by our data, γδ17 T cells expressing low intracellular glutathione are particularly susceptible to oxidative suppression, whereas CD8^+^ T cells likely require greater ROS concentrations. Interestingly, Treg cells were also recently shown to be highly sensitive to oxidative stress in the tumor microenvironment, due in this case to a weak nuclear factor (erythroid-derived 2)-like 2 (NRF2)-associated antioxidant system [59], which may explain our observation of reduced Treg accumulation (Fig 1C).

On the other hand, the pleiotropic roles of neutrophils may be associated with heterogeneous maturation and activation phenotypes in different tumor models as well as mouse backgrounds (such as FVB versus C57BL/6) [46]. For example, KEP tumor-induced neutrophils were immature and expressed c-kit protein and S100 calcium-binding protein A8 (s100a8) transcript, which are molecules associated with pro-metastatic features [11]; in contrast, peritoneal B16 tumor–induced neutrophils did not express c-kit or up-regulated s100a8 when compared to neutrophils from the bone marrow of tumor-free mice. Moreover, our data on *Genista* mice, which lack mature neutrophils, indicate that it is the mature neutrophils that suppress γδ17 T cells. Thus, we propose that neutrophils can be suppressive and yet antitumoral by targeting γδ17 T cells, which is in line with their protective role, linked to IL-17 inhibition, in the murine Lewis lung carcinoma model [60].

In humans, Vδ1^+^ T cells can be important IL-17 producers that favor cancer progression through induction of inflammation [16] and recruitment of immunosuppressive myeloid cells [13]. Consistent with our mouse data, we found that Vδ1^+^ T cells express low basal levels of glutathione and are highly susceptible to ROS. In line with this, human neutrophils from healthy donors have also been shown to impact circulating γδ T-cell activation and cytokine production and proliferation through production of ROS [61].

ROS are short-lived molecules that originate from molecular oxygen and include superoxide (O_2_^−^), hydrogen peroxide (H_2_O_2_), hypochlorous acid (HCl), and hydroxyl radical, among others. Superoxide and hydrogen peroxide are the most common ROS involved in biological processes. Superoxide is rapidly dismutated to hydrogen peroxide or immediately reacts with surrounding molecules; hydrogen peroxide is more stable and can diffuse in the microenvironment and across cell membranes [62]. As it is technically challenging to pinpoint which species acts on γδ17 T cells in vivo, we favor hydrogen peroxide but cannot exclude a role for other ROS species such as hypochlorous acid, which is produced from hydrogen peroxide by myeloperoxidase (highly expressed by neutrophils) [63].

In conclusion, our study identifies neutrophil-derived ROS as important regulators of pro-tumoral γδ17 T cells that express particularly low levels of the antioxidant glutathione, which may open new avenues for clinical translation. On the other hand, it challenges the widely accepted view of immunosuppressive myeloid cells solely as being detrimental in cancer progression. In fact, additional lines of evidence support antitumor functions of neutrophils [64], including enhanced cytotoxic activity [52,60,65]. Importantly, neutrophils appear to contribute to the efficacy of rituximab and trastuzumab treatments [66–68], Bovis bacillus Calmette-Guerin treatment in bladder cancer [69], radiotherapy [70], and chemotherapy [65]. Therefore, we strongly believe that the pleiotropic functions of neutrophils can be manipulated—in order to boost their protective activities—in future cancer immunotherapy approaches.

## Materials and methods

### Ethics statement

Buffy coats from healthy volunteers were obtained under the agreement (15.12.2003) between Instituto de Medicina Molecular (iMM) and Instituto Português do Sangue e da Transplantação and were approved by the local ethical committee (Centro de Ética do Centro Hospitalar Lisboa Norte—Hospital de Santa Maria). All mouse experiments performed in this study were evaluated and approved by our institutional ethical committee (iMM-Orbea) and the national competent authority (DGAV) under the license number 019069. Briefly, euthanasia was performed by CO_2_ inhalation. Anesthesia was performed by isofluorane inhalation or by intraperitoneal administration of ketamine and medetomidine, and reversed by administration of atipamezole.

### Mice and tumor cell lines

C57Bl/6J (B6) WT mice and B6.TCRα^−/−^ and B6.TCRδ^−/−^ mice were purchased from Charles River Laboratories. B6.*Il17*^−/−^ mice were kindly provided by Fiona Powrie (University of Oxford, Oxford, United Kingdom), with permission from Yoichiro Iwakura (Tokyo University of Science, Chiba, Japan). *Genista* mice were imported from the Center of Immunology Marseille Luminy (France) and bred in house. *Genista* homozygous mice were used as a neutropenic model and were compared to their heterozygous littermate controls. Mice were maintained in specific pathogen-free facilities of iMM. *Cybb*^−/−^ male mice and their respective C57Bl/6J controls were purchased from Jackson laboratories and maintained in specific pathogen-free facilities at the Francis Crick Institute. IFN-γ/IL-17 double-reporter mice, generated by crossing IFN-γ-YFP mice [71] with Il17a-GFP mice [72], were used to sort IL-17+ and IFN-γ+ γδ T cells from lymph nodes. Animals were 5–13 weeks of age and aged-matched within 3 weeks, and no randomization or blinding was performed when mice were allocated into experimental groups. Mice that did not develop visible tumors were excluded from the analysis. The Hepa 1–6 murine hepatocellular carcinoma cell line and B16-F0 melanoma cell line were purchased from ATCC (Manassas, VA). Cells were tested for mycoplasma contamination and maintained in Dulbecco’s Modified Eagle Medium (DMEM) with 10% (vol/vol) FCS (Gibco; Thermo Fisher Scientific) and 1% (vol/vol) penicillin/streptomycin (Sigma/Merck). Lentiviral infection of Hepa 1–6 cells with luciferase reporter was performed as previously described [73].

### In vivo tumor transplantation and treatments

For orthotopic hepatocellular carcinoma model, anesthetized mice received 1 × 10^6^ Hepa 1–6 cells implanted intrahepatically in 20 μL PBS through surgical procedure. Mice were euthanized 2–3 weeks later, and tumors were extracted for subsequent analysis. We injected 5 × 10^4^ B16-F0 tumor cells intraperitoneally in 100 μL of PBS. Tumor growth was evaluated in situ by bioluminescence imaging as previously described [73]. For proliferation analysis, mice received 1.5 mg of BrdU i.p. at day 4 post–tumor inoculation and then were fed daily with 0.8 mg/mL BrdU (Sigma/Merck) in drinking water until the indicated day of analysis. For the ID8 and B16 co-injection experiment, 1 × 10^6^ ID8 cells were injected intraperitoneally. ID8 tumors were let to grow for 2 weeks, after which 5 × 10^4^ B16 cells were inoculated i.p. One group of mice was injected with anti-Gr-1 as described below. Two weeks after B16 tumor inoculation (and 4 weeks upon ID8 injection), mice were euthanized and peritoneal exudate cells analyzed by FACS.

For in vivo antibody depletion, 70 μg anti-Gr1 (B16 intraperitoneal model), 250 μg (Hepa 1–6 intrahepatic model) (Bio X Cell, clone RB6-8C5), 250 μg anti-Ly6G antibody (Bio X Cell, clone 1A8), 1 mg anti-CD25 (clone PC-61.5.3, kindly provided by Luis Graça [iMM]), 300 μg anti-CD115 (Bio X Cell, clone AFS98), 70 μg isotype control (Bio X Cell, LTF-2), or PBS was injected i.p. at days 4, 8, and 12 post–tumor inoculation. For monocyte/macrophage depletion, 100 μL of clodronate liposomes (Liposoma B.V.) were injected s.c. or i.v. at days 4, 8, and 12 post–tumor inoculation.

NAC (Sigma/Merck) was resuspended in PBS (pH = 7) and administrated i.p. every other day, from day 4 post–tumor injection, at a concentration of 15 mg/kg.

Fingolimod (FTY720, Sigma/Merck) was given in the drinking water (2.5 μg/mL) from day 4 post–tumor inoculation.

### Human samples

Blood leukocytes (buffy coat cells) were isolated by gradient centrifugation in Histopaque and each lymphocyte population was FACS-sorted in FACS Aria (BD Biosciences).

### Cell preparation, cell sorting, and flow cytometry and analysis

Hepa 1–6 tumors were harvested, finely chopped, and digested with 1 mg/mL collagenase Type I, 0.4 mg/mL collagenase Type IV (Worthington), and 10 μg/mL DNase I (Sigma/Merck) for 30 minutes at 37 °C. Cell suspension was then filtered through a 100 μm nylon cell strainer (Falcon/Corning). Peritoneal exudate cells were obtained from the lavage of the peritoneal cavity with 5 mL ice-cold DMEM with 10% (vol/vol) FCS. Erythrocytes were osmotically lysed using RBC Lysis Buffer (Biolegend). For surface staining, cells were Fc blocked with anti-CD16/32 (93; eBioscience/Thermo Fisher Scientific) and incubated for 45 minutes with antibodies and LIVE/DEAD Fixable Near-IR (Thermo Fisher Scientific) in complete RPMI medium. The following monoclonal antibodies were purchased from eBioscience/Thermo Fisher Scientific: anti-CD3ε (clone; 145-2C11), anti-CD4 (RM4-5), anti-CD11b (M1/70), anti-F4/80 (BM8), anti-MHC II (M5/114.15.2), anti-CD27 (LG.7F9), and anti-TCRγ4 (UC3-10A6); from Biolegend: anti-CD8α (53–6.7), anti-CD45 (30-F11), anti-TCRδ (GL3), anti-Ly6C (HK1.4), anti-Ly6G (1A8), anti-NK1.1 (PK136), and anti-TCRγ1 (2.11); and from BD Pharmigen: anti-CD44 (IM7).

For T-cell intracellular cytokine staining, cells from tumor, PEC, or spleen were stimulated with 50 ηg/mL phorbol 12-myristate 13-acetate (PMA; Sigma/Merck) and 1 μg/mL ionomycin (Sigma/Merck) for 3 hours at 37 °C in the presence of 10 μg/mL brefeldin-A (Sigma/Merck) and 2 μM monensin (eBioscience/Thermo Fisher Scientific). Cells were fixed and permeabilized using the Foxp3/Transcription Factor Staining Buffer set (eBioscience/Thermo Fisher Scientific), following the manufacturer’s instructions, and then incubated for 30 minutes at room temperature, with the following antibodies from eBioscience/Thermo Fisher Scientific: anti-IFN-γ (XMG1.2), anti-IL-17 (TC11-18H10.1), Foxp3 (FJK-16s), and Ki67 (16A8). For BrdU staining, FITC BrdU Flow Kit (BD Pharmingen) was used following manufacturer’s instructions. For TCRγ6 (Vγ6) detection, staining with GL3 and 17D1 monoclonal antibodies (kind gift from Prof. Adrian Hayday, The Francis Crick Institute, UK) was performed as previously described [29]. For Annexin V staining, Annexin V Kit (eBioscience/Thermo Fisher Scientific) was used following manufacturer’s instructions. Cell Event Caspase 3/7 Green (from Thermo Fisher Scientific) was used according to manufacturer’s instructions. For superoxide detection, cells were stained with dihydroethidium (Thermo Fisher Scientific) at a final concentration of 100 μM in PBS for 45 minutes at 37 °C.

Cells were acquired on a FACS Fortessa (BD Biosciences) or LSR II, sorted on FACS Aria, and data analyzed using FACS Diva or FlowJo software (Tree Star).

### In vitro γδ T-cell stimulation and inhibition

Lymphoid (spleen and lymph nodes) were harvested from C57Bl/6J or B6.TCRα^−/−^ mice. Cell suspensions were stained with anti-CD3ε (145-2C11), anti-TCRδ (GL3), and anti-CD27 (LG.7F9) for 30 minutes at room temperature. CD27^+^, CD27^−^ γδ T cells, CD4, and CD8 T cells were FACS-sorted and stained with 1 mM of Cell Trace Violet (Thermo Fisher Scientific) in PBS for 20 minutes at room temperature. Cells were incubated on plate-bound anti-CD3ε (145.2C11) (10 μg/mL) plus anti-CD28 mAb (37.51) (5 μg/mL) in the presence of IL-7 (50 ηg/mL) for 72 hours. IL-7 was from Peprotech and the antibodies were from eBiosciences/Thermo Fisher Scientific or BioLegend.

Neutrophils were isolated from the peritoneal exudates of B16 tumor–bearing WT or *Cybb*^−/−^ mice or from the bone marrow of tumor-free or tumor-bearing WT mice. For neutrophil purification, cells were stained with αGr-1–PE (RB6-8C5) at a concentration of 40 ηg/mL, and mouse anti-PE selection kit (STEMCELL Technologies) was used.

Alternatively, cells were cultured with different concentrations of H_2_O_2_, the superoxide-generating system xanthine/xanthine oxidase (Sigma/Merck), or catalase (Sigma/Merck).

Human cells were cultured with soluble anti-CD3 (clone HIT3a, 1 μg/mL) and IL-2 (10 ηg/mL) for 6 days in the presence of 100–300 μM of H_2_O_2_. CTV dilution was assessed by FACS.

### RNA isolation, cDNA production, and real-time PCR

mRNA was prepared from FACS-sorted cell populations using High Pure RNA Isolation kit (Roche). Reverse transcription was performed with random oligonucleotides (Invitrogen) using Moloney murine leukemia virus reverse transcriptase (Promega) for 1 hour at 42 °C. Relative quantification of specific cDNA species to endogenous reference *hprt* or *β2microglobulin* was carried out using SYBR on ABI ViiA7 cycler (Applied Biosystems). The C_T_ for the target gene was subtracted from the C_T_ for endogenous references, and the relative amount was calculated as 2^−ΔCT^. Primer sequences were the following: *nfe2l2* forward, GCAGCCATGACTGATTTAAGC, *nfe2l2* reverse, CAGCCAGCTGCTTGTTTTC, *gclc* forward, GGCTCTCTGCACCATCACTT, *gclc* reverse, GTTAGAGTACCGAAGCGGGG, *gclm* forward, AGGAGCTTCGGGACTGTATCC, *gclm* reverse, GGGACATGGTGCATTCCAAAA, *gpx1* forward, CAATGTAAAATTGGGCTCGAA, *gpx1* reverse, GTTTCCCGTGCAATCAGTTC, *gpx4* forward, TAAGAACGGCTGCGTGGT, *gpx4* reverse, GTAGGGGCACACACTTGTAGG, *gsr* forward, ATCGTGCATGAATTCCGAGT, *gsr* reverse, GGTGGTGGAGAGTCACAAGC, *gss* forward, CACTATCTCTGCCAGCTTTGG, *gss* reverse, TTATTCAGGACATTGAGAACGTG, *mdh2* forward, TGACCTGTTCAACACCAACG, *mdh2* reverse, GATGGGGATGGTGGAGTTC, *pgd* forward, ATGGCCCAAGCTGACATTG, *pgd* reverse, GCACAGACCACAAATCCATGAT, *prdx1* forward, GTTGGCCGCTCTGTGGATGAGAT, *prdx1* reverse, ATCACTGCCAGGTTTCCAGCCAGC, *prdx2* forward, GTTCTCCGGCCTAGGGCTCTCTC, *prdx2* reverse, GCCGGAGGCCATGACTGCGTG, *prdx3* forward, GAACCTGTTTGACAGACATACTGTG, *prdx3* reverse, GGGGTGTGGAAAGAGGAACT, *prdx4* forward, CTCAAACTGACTGACTATCGTGG, *prdx4* reverse, CGATCCCCAAAAGCGATGATTTC, *sod1* forward, TACTGATGGACGTGGAACCC, *sod1* reverse, GAACCATCCACTTCGAGCA, *srxn1* forward, AGTAGTAGTCGCCACCCTGG, *srxn1* reverse, AGAGCCTGGTGGACACGAT, *txn1* forward, TGCTACGTGGTGTGGACCTTGC, *txn1* reverse, TCTGCAGCAACATCCTGGCAGT, *txn2* forward, CGACCTTTAACGTCCAGGATG, *txn2* reverse, ACTGTGCATGAAAGTCCACAAC, *txnrd1* forward, ATGGACAGTCCCATCCCGGGA, *txnrd1* reverse, GCCCACGACACGTTCATCGTCT, *txnrd3* forward, CCAAGAAATATGGCTGGGAGT, *txnrd3* reverse, TGTAGCCCCAGTTCAAGGAG.

### H_2_O_2_, protein oxidation and glutathione quantification

H_2_O_2_ was measured using OxiSelect Hydrogen Peroxide/Peroxidase Assay Kit (Cell Biolabs), following manufacturer’s instructions. Protein oxidation was measured by flow cytometry using the FlowCellect oxidative stress kit (Sigma/Merck), following manufacturer’s instructions. For glutathione quantification, cells were FACS-sorted from spleen and LN of C57Bl/6J or B6.TCRα^−/−^ tumor-free mice and lysed in 5% metaphosphoric acid at a concentration of 2 × 10^6^ cells per mL (for smaller cell numbers, the volume was adjusted accordingly). Glutathione (GSSG/GSH) detection kit (Enzo Life Sciences) was used to quantify total glutathione according to manufacturer’s instructions.

### Statistical analysis

No statistical methods were used to predetermine sample size. Statistics were done using nonparametric two-tailed Mann-Whitney test or, if both groups followed a normal distribution (tested by D’Agostino and Pearson normality test), using two-tailed unpaired Student *t* test with 95% confidence intervals for unrelated samples. For paired samples, Wilcoxon-matched-pairs test was used. When more than two groups were compared, two-way ANOVA followed by Tukey HSD post hoc test was performed. Unless otherwise indicated, individual values and mean are plotted, or mean ± SEM. *p < 0.05; **p < 0.01; ***p < 0.001.

## Supporting information

S1 Data Underlying data for Figs 1–4 and S1–S5 Figs. (XLSX)

S1 Fig Leukocyte frequencies in the peritoneal cavity upon B16 and/or ID8 tumor challenge. **(A)** Neutrophil frequencies in peritoneal exudates of B16 tumor–bearing or ID8 tumor–bearing mice, assessed at week 2 (B16) or at weeks 2 and 8 (ID8) after tumor implantation. **(B)** IL-17^+^ γδ, CD27^+^ γδ T-cell frequency in peritoneal exudates of tumor-free, ID8 + B16 tumor–bearing and ID8 + B16 tumor–bearing mice depleted for neutrophils. Statistical analysis was performed by Mann-Whitney test.
(TIF)

S2 Fig Characterization of models for leukocyte ablation in vivo. **(A)** Neutrophil, monocyte, and Treg cell frequencies in peritoneal exudates of B16 tumor–bearing mice treated with αGR-1, αLy6G, αCD115 + clodronate–containing liposomes and αCD25 mAbs. **(B)** Representative FACS plots of neutrophils and summary of Ly6G and CD11b MFI in neutrophils from *Genista* heterozygous or homozygous Hepa 1–6 tumor–bearing mice. mAb, monoclonal antibody; MFI, mean fluorescence intensity; Treg, regulatory T.
(TIF)

S3 Fig Tumor-associated γδ T cells are the main source of IL-17 upon neutrophil depletion. **(A)** Representative FACS plots and frequency of IL-17^+^ cells and IFN-γ^+^ cells in the peritoneal exudates of B16 tumor–bearing (top) and Hepa 1–6 tumor–bearing mice (bottom), either in the presence (Neu +) or absence (Neu −) of neutrophils. Red and blue circles represent αGr-1 mAb-treated or PBS-treated C57BL/6 mice, respectively, whereas red and blue triangles represent *Genista* homozygous or littermate controls, respectively. Data were pooled from three independent experiments. **(A)** Representative FACS plots and summary chart of γδ T-cell and CD4^+^ T-cell contributions to the IL-17^+^ CD3^+^ pool, as well as their MFI in the absence of neutrophils (as in **A**) or in intraperitoneal B16 (top) or intrahepatic Hepa 1–6 (bottom) tumor models. Data were pooled from two independent experiments. Dotted lines link subsets from the same mouse. Statistical analysis was performed using Mann-Whitney test or Wilcoxon-matched-pairs signed rank test (for IL-17 MFI analysis).
(TIF)

S4 Fig Neutrophils do not impact apoptosis or recruitment of Vγ6^+^ T cells. **(A)** Apoptotic Vγ6^+^ T cells, assessed by annexin V and caspase 3/7 cleavage, in the peritoneal exudates of PBS or aGr-1 mAb-treated B16 tumor–bearing mice at days 9 and 13 post–tumor inoculation. Data were pooled from two independent experiments. **(B)** Frequency of Vγ6^+^, CD8^+^, and CD4^+^ T cells in the peritoneal exudates of PBS or αGr-1 mAb-treated or FTY720-treated PBS or αGr-1 mAb-treated B16 tumor–bearing mice. Statistical analysis was performed using two-way ANOVA followed by Tukey HSD post hoc test.
(TIF)

S5 Fig CD27^−^ γδ T cells are highly susceptible to H_2_O_2_-dependent suppression by neutrophils. **(A)** In vitro inhibition of CD27^−^ γδ, CD27+ γδ, CD4, and CD8 T-cell proliferation in the presence of neutrophils from the peritoneal cavity of B16 tumor–bearing mice. **(B)** CD27^−^ γδ T-cell proliferation cultured alone, in the presence of neutrophils from the peritoneal cavity of B16 tumor–bearing mice, with or without catalase.
(TIF)

## Abbreviations

BrdU: 5-bromodeoxycytidine
CTV: cell trace violet
Cxcr2: C-X-C chemokine receptor type 2
*Cybb*: cytochrome B(−245), β subunit
DMEM: Dulbecco’s Modified Eagle Medium
DNP: dinitrophenyl
FACS: Fluorescence-activated cell sorting
FSC: forward scatter
FTY720: fingolimod
Gcl: glutamate-cysteine ligase
Gclc: glutamate-cysteine ligase catalytic subunit
Gclm: glutamate-cysteine ligase modifier subunit
Gfi1: growth factor independence 1
Gpx: glutathione peroxidase
Gr1: granulocyte marker
GSH: glutathione
Gsr: glutathione reductase
Gss: glutathione synthetase
*Hprt*: hypoxanthine-guanine phosphoribosyltransferase
IFN-γ: interferon gamma
IL-17: interleukin 17
IL-6: interleukin 6
iNOS: inducible nitric oxide synthase
Itgb2: integrin beta 2
KEP: K14cre;Cdh1F/F;Trp53F/F
Lipo: clodronate liposome
Ly6G: lymphocyte antigen 6 complex locus G6D
mAb: monoclonal antibody
Mdh2: malate dehydrogenase 2
MFI: mean fluorescence intensity
MMP-9: matrix metallopeptidase 9
NAC: N-acetylcysteine
NOX2: NADPH oxidase 2
NRF2: nuclear factor (erythroid-derived 2)-like 2
PBS: phosphate buffered saline
PEC: peritoneal exudate cell
Pgd: phosphogluconate dehydrogenase
Prdx: peroxiredoxin
RLU: relative luminescence units
ROS: reactive oxygen species
s100a8: S100 calcium-binding protein A8
*Sod1*: superoxide dismutase 1
*Srxn1*: sulfiredoxin-1
SSC: side scatter
STAT3: signal transducer and activator of transcription 3
TCR: T-cell receptor
TNF-α: tumor necrosis factor alpha
Treg: regulatory T
Txn: thioredoxin
Txnrd: thioredoxin reductase
VEGF: vascular endothelial growth factor
WT: wild-type
X/XO: xanthine/xanthine oxidase
γδ17: IL-17–producing γδ T cells
γδIFN-γ: IFN-γ–producing γδ T cell
