# Supplementary figures and images for: Tumor-associated neutrophils suppress pro-tumoral IL-17+ γδ T cells through induction of oxidative stress
Source: PLoS Biol. 2018 May 11;16(5):e2004990. doi: 10.1371/journal.pbio.2004990 (PMC5965901; doi:10.1371/journal.pbio.2004990)

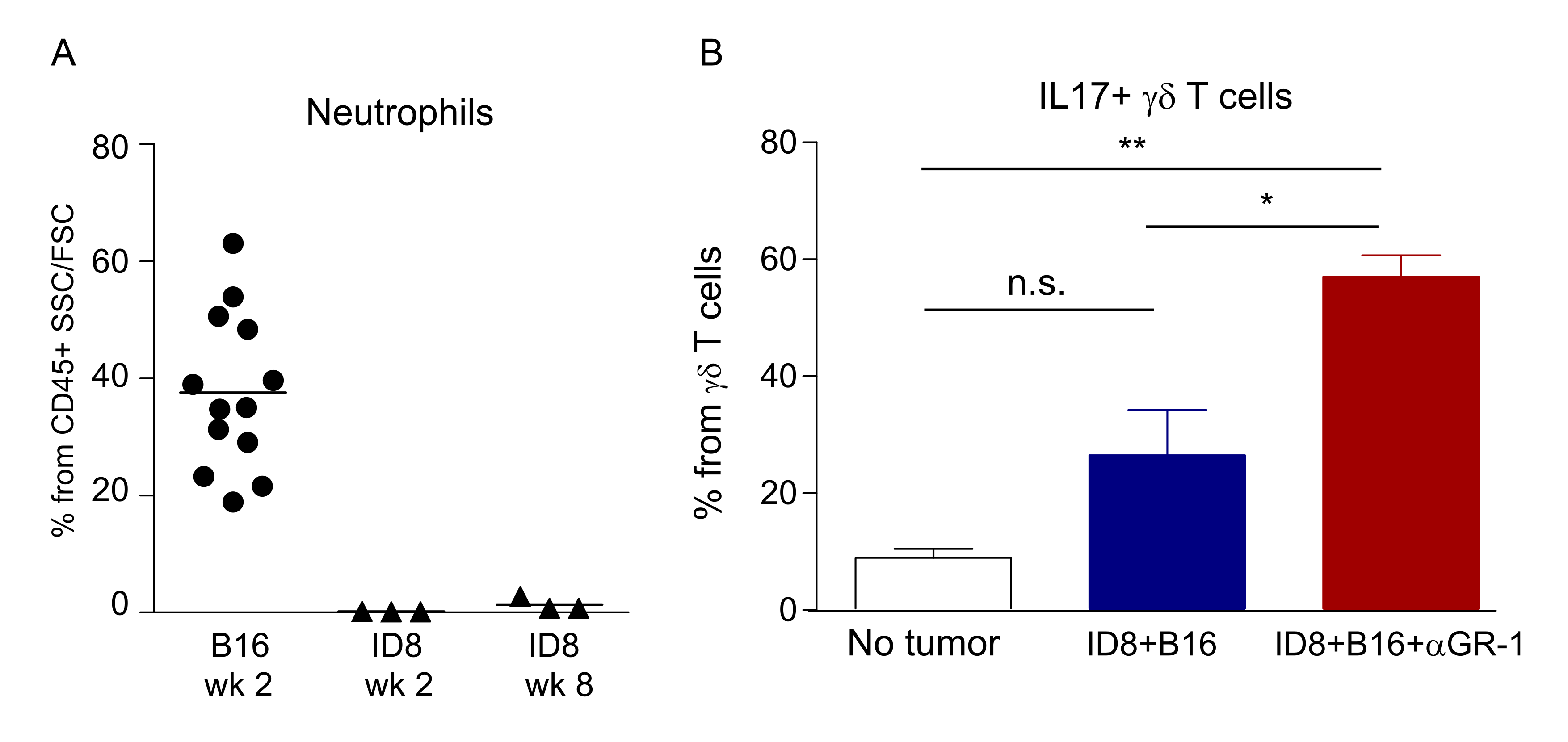

Supplement: S1 Fig — (A) Neutrophil frequencies in peritoneal exudates of B16 tumor–bearing or ID8 tumor–bearing mice, assessed at week 2 (B16) or at weeks 2 and 8 (ID8) after tumor implantation. (B) IL-17+ γδ, CD27+ γδ T-cell frequency in peritoneal exudates of tumor-free, ID8 + B16 tumor–bearing and ID8 + B16 tumor–bearing mice depleted for neutrophils. Statistical analysis was performed by Mann-Whitney test. (TIF) [file pbio.2004990.s002.tif]

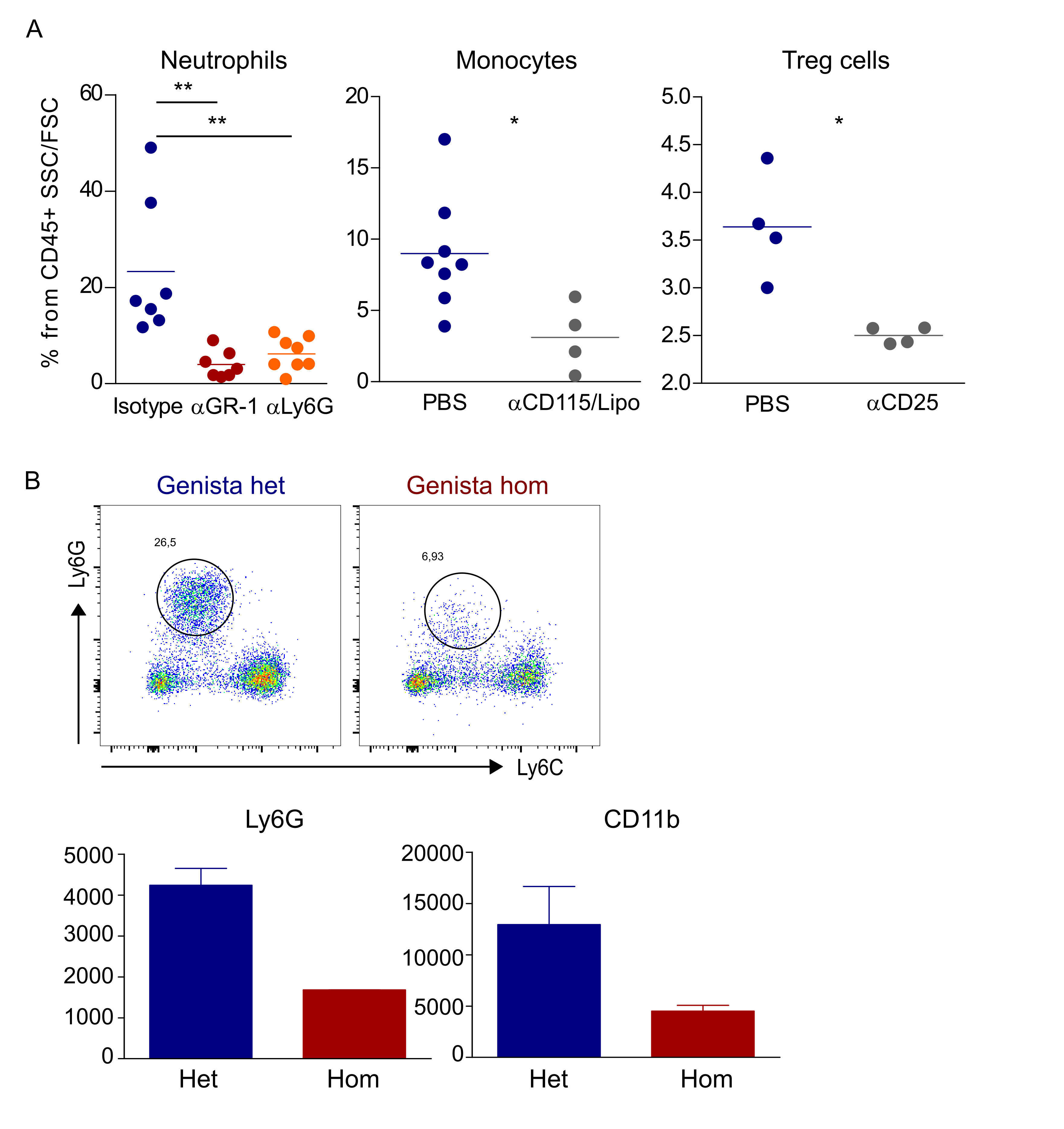

Supplement: S2 Fig — (A) Neutrophil, monocyte, and Treg cell frequencies in peritoneal exudates of B16 tumor–bearing mice treated with αGR-1, αLy6G, αCD115 + clodronate–containing liposomes and αCD25 mAbs. (B) Representative FACS plots of neutrophils and summary of Ly6G and CD11b MFI in neutrophils from Genista heterozygous or homozygous Hepa 1–6 tumor–bearing mice. mAb, monoclonal antibody; MFI, mean fluorescence intensity; Treg, regulatory T. (TIF) [file pbio.2004990.s003.tif]

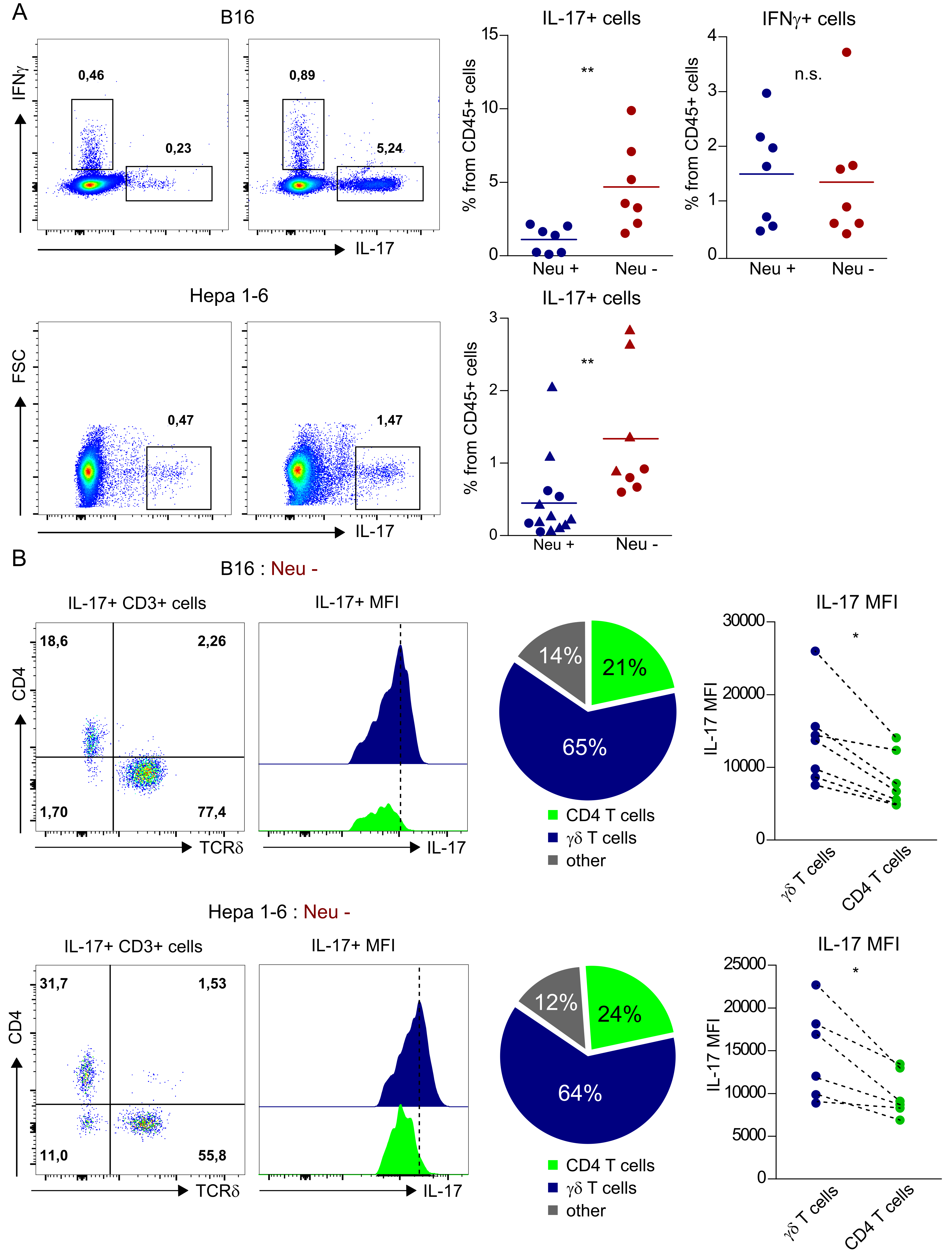

Supplement: S3 Fig — (A) Representative FACS plots and frequency of IL-17+ cells and IFN-γ+ cells in the peritoneal exudates of B16 tumor–bearing (top) and Hepa 1–6 tumor–bearing mice (bottom), either in the presence (Neu +) or absence (Neu −) of neutrophils. Red and blue circles represent αGr-1 mAb-treated or PBS-treated C57BL/6 mice, respectively, whereas red and blue triangles represent Genista homozygous or littermate controls, respectively. Data were pooled from three independent experiments. (A) Representative FACS plots and summary chart of γδ T-cell and CD4+ T-cell contributions to the IL-17+ CD3+ pool, as well as their MFI in the absence of neutrophils (as in A) or in intraperitoneal B16 (top) or intrahepatic Hepa 1–6 (bottom) tumor models. Data were pooled from two independent experiments. Dotted lines link subsets from the same mouse. Statistical analysis was performed using Mann-Whitney test or Wilcoxon-matched-pairs signed rank test (for IL-17 MFI analysis). (TIF) [file pbio.2004990.s004.tif]

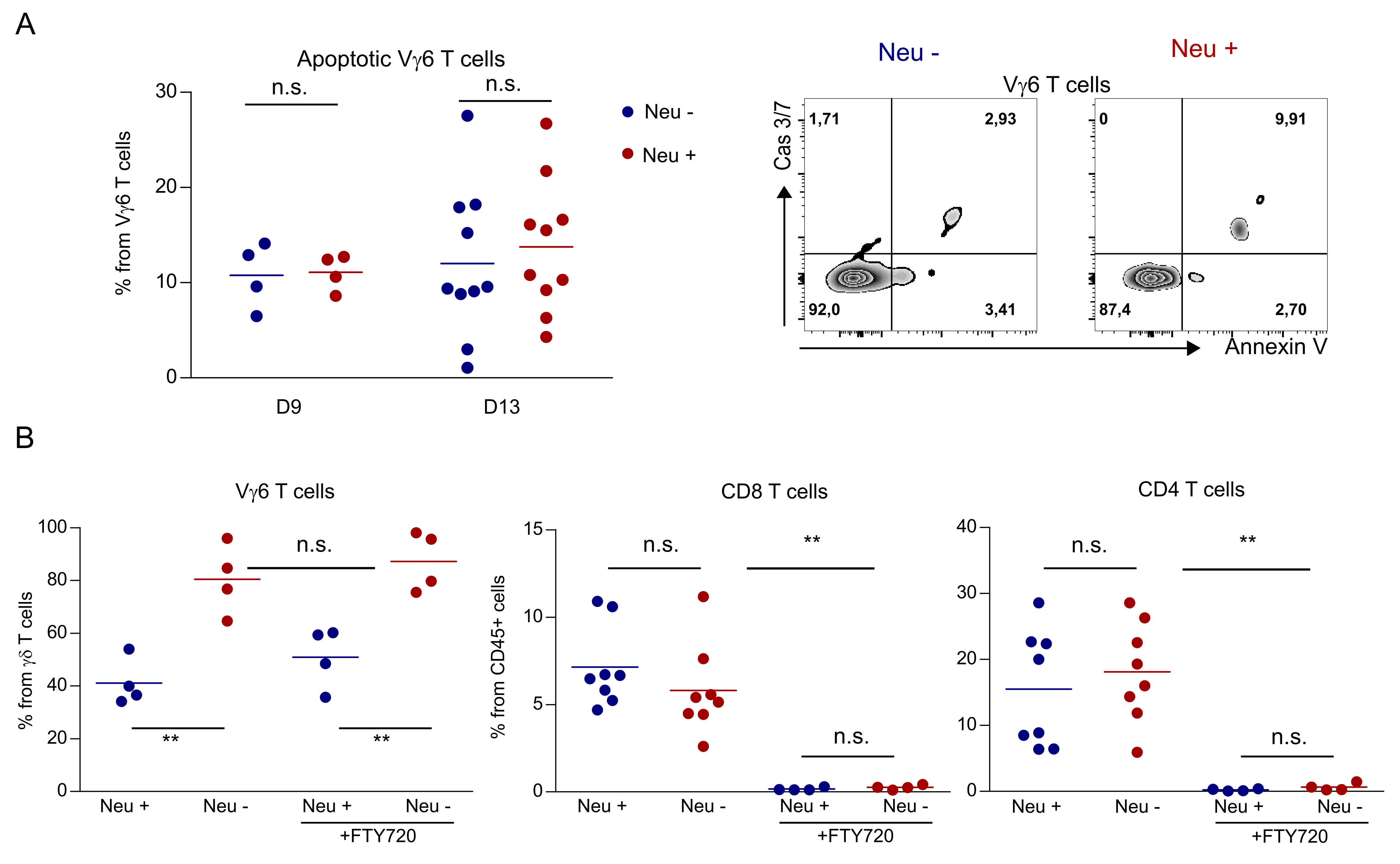

Supplement: S4 Fig — (A) Apoptotic Vγ6+ T cells, assessed by annexin V and caspase 3/7 cleavage, in the peritoneal exudates of PBS or aGr-1 mAb-treated B16 tumor–bearing mice at days 9 and 13 post–tumor inoculation. Data were pooled from two independent experiments. (B) Frequency of Vγ6+, CD8+, and CD4+ T cells in the peritoneal exudates of PBS or αGr-1 mAb-treated or FTY720-treated PBS or αGr-1 mAb-treated B16 tumor–bearing mice. Statistical analysis was performed using two-way ANOVA followed by Tukey HSD post hoc test. (TIF) [file pbio.2004990.s005.tif]

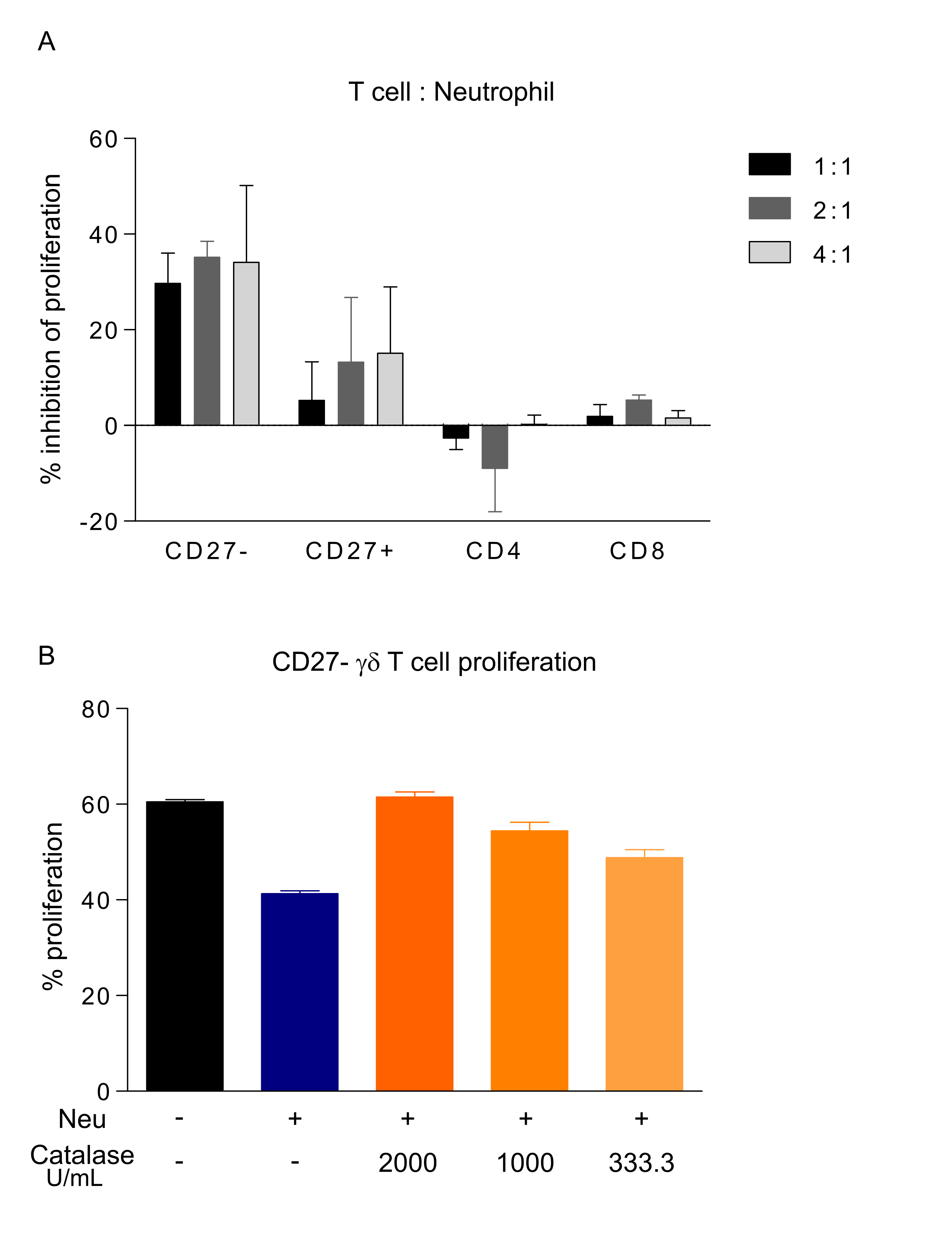

Supplement: S5 Fig — (A) In vitro inhibition of CD27− γδ, CD27+ γδ, CD4, and CD8 T-cell proliferation in the presence of neutrophils from the peritoneal cavity of B16 tumor–bearing mice. (B) CD27− γδ T-cell proliferation cultured alone, in the presence of neutrophils from the peritoneal cavity of B16 tumor–bearing mice, with or without catalase. (TIF) [file pbio.2004990.s006.tif]
